# A pragmatic harm reduction approach to manage a large outbreak of wound botulism in people who inject drugs, Scotland 2015

**DOI:** 10.1186/s12954-018-0243-9

**Published:** 2018-07-11

**Authors:** Kirsten M. A. Trayner, Amanda Weir, Andrew McAuley, Gauri Godbole, Corinne Amar, Kathie Grant, Gillian Penrice, Kirsty Roy

**Affiliations:** 1 Health Protection Scotland, NHS National Services Scotland, Meridian Court, 5 Cadogan Street, Glasgow, Scotland; 2 Glasgow Caledonian University, Cowcaddens Road, Glasgow, Scotland; 3 Gastrointestinal Bacteria Reference Unit (GBRU), National Infection Service, Public Health England, London, England; 4 Public Health Protection Unit, Gartnavel Royal Hospital, NHS Greater Glasgow and Clyde, Glasgow, Scotland

**Keywords:** Wound botulism, People who inject drugs, Outbreak investigation, Harm reduction

## Abstract

**Background:**

People who inject drugs (PWID) are at an increased risk of wound botulism, a potentially fatal acute paralytic illness. During the first 6 months of 2015, a large outbreak of wound botulism was confirmed among PWID in Scotland, which resulted in the largest outbreak in Europe to date.

**Methods:**

A multidisciplinary Incident Management Team (IMT) was convened to conduct an outbreak investigation, which consisted of enhanced surveillance of cases in order to characterise risk factors and identify potential sources of infection.

**Results:**

Between the 24th of December 2014 and the 30th of May 2015, a total of 40 cases were reported across six regions in Scotland. The majority of the cases were male, over 30 and residents in Glasgow. All epidemiological evidence suggested a contaminated batch of heroin or cutting agent as the source of the outbreak. There are significant challenges associated with managing an outbreak among PWID, given their vulnerability and complex addiction needs. Thus, a pragmatic harm reduction approach was adopted which focused on reducing the risk of infection for those who continued to inject and limited consequences for those who got infected.

**Conclusions:**

The management of this outbreak highlighted the importance and need for pragmatic harm reduction interventions which support the addiction needs of PWID during an outbreak of spore-forming bacteria. Given the scale of this outbreak, the experimental learning gained during this and similar outbreaks involving spore-forming bacteria in the UK was collated into national guidance to improve the management and investigation of future outbreaks among PWID.

## Background

Wound botulism is a serious and potentially fatal acute paralytic illness caused by a highly potent neurotoxin produced by *Clostridium botulinum* [1]. Spores of *C. botulinum* are found in soil and can be introduced into illicit drugs such as heroin at any stage from point of production to use [1, 2]. People who inject drugs (PWID) are at an increased risk of infection, particularly if they inject under the skin (“skin popping”) or into muscle (“muscle popping”) either deliberately or as a result of a “missed hit” (i.e. someone missing a vein when trying to inject intravenously). The necrotic devitalised tissue at the site of injection provides an ideal anaerobic environment for *C. botulinum* spores to germinate, multiply and produce toxin which then gets absorbed into the systemic circulation causing a neuroparalytic syndrome [3–7].

Although wound botulism in PWID have been noted in numerous countries across the world [4, 8–12], the UK appears to be disproportionately affected with a number of outbreaks of wound botulism and other spore-forming bacteria such as *Clostridium novyi*, *Bacillus anthracis* and *Clostridium tetani* [5, 13–17]*.* Between 2000 and 2014, there were a total of 170 reported cases of wound botulism among PWID in the UK. In 2015 alone, there were 47 reported cases, the largest outbreak of wound botulism seen among PWID in Europe, which was focused on Glasgow, Scotland [18]. Prior to the outbreak, the last probable (though unconfirmed) case in Scotland was reported in 2009.

The outbreak began on Wednesday the 24th of December 2014, when NHS Greater Glasgow and Clyde (NHS GGC) health board Public Health Protection Unit (PHPU) were notified that a Glasgow resident in their thirties with a history of injecting drug use had been admitted to a hospital in Glasgow with neurological symptoms suggestive of botulism. Exactly 1 week later, NHS GGC were notified of a further suspected case, also in their thirties with a history of injecting drug use. The identification of two probable cases of wound botulism with a history of injecting drugs in a 1-week period suggested the contamination of a batch of heroin and/or cutting agent. In the ensuing weeks, further cases of suspected wound botulism were reported across six NHS health boards in Scotland. This report describes the epidemiological characteristics of what became the largest outbreak of wound botulism among PWID seen in Europe thus far [18], and the pragmatic harm reduction approach taken to limit morbidity and mortality associated with the infection during the outbreak. This manuscript aims to raise awareness of wound botulism and the importance and the need of harm reduction policies for the management of outbreaks among PWID.

## Methods

### Outbreak response

Initially, the outbreak was managed by an Incident Management Team (IMT) based at NHS GGC. As the number of NHS health boards affected increased, a national IMT was convened and led by Health Protection Scotland (HPS). The multidisciplinary IMT consisted of representatives from NHS health boards, police (Police Scotland), non-governmental organisations (Scottish Drugs Forum), Public Health England (PHE), reference laboratories (Gastrointestinal Bacteria Reference Unit (GBRU)) and the Scottish Government.

### Epidemiologic investigation

The IMT adapted the European Centre for Disease Control (ECDC) case definition of botulism for the outbreak, which included clinical, epidemiological and microbiological criteria (available from: http://eur-lex.europa.eu/legal-content/EN/TXT/PDF/?uri=CELEX:32012D0506&qid=1428573336660&from=EN#page=7) (Table 1). The case definition was categorised by the degree of certainty regarding the diagnosis as:

- *Probable case*—a high clinical suspicion and epidemiological evidence consistent with botulism
- *Confirmed case*—clinical, epidemiological and microbiological evidence consistent with botulism

**Table 1:** *Clostridium botulinum* outbreak case definition

Clinical	Any person with at least one of bilateral cranial nerve impairment (e.g. diplopia, blurred vision, dysphagia, bulbar weakness) or peripheral symmetric paralysis.
Epidemiological	Use of illicit drugs by any route within the 2 weeks prior to onset of symptoms.
Microbiological	Isolation of *Clostridium botulinum* from infected wound and/or detection of botulinum toxin in a clinical specimen.

Additionally, a “possible” case classification was used, when dealing with the initial report of suspected cases that merited further investigation. This provisional classification was given following review of the case by the Consultant Microbiologist of the GBRU and the treating physician. Cases were classified as “probable” or “confirmed” when further information became available.

Botulism is legally notifiable under the Public Health Act (Scotland) 2008 (available from: http://www.gov.scot/Resource/0039/00398162.pdf). For all cases identified, information on personal and medical details, source of drugs and drug use prior to the onset of illness, was collected using an established national enhanced surveillance form (available from: https://www.gov.uk/government/uploads/system/uploads/attachment_data/file/577402/PWID_Botulism_Questionnaire_2006.1.6_14_December_2016.pdf). Copies of the completed form were sent to HPS and collated on a copy of an Access database utilised by PHE for the UK surveillance of botulism associated with PWID.

### Laboratory investigations

Microbiological confirmation of a clinical diagnosis of botulism requires the timely collection of appropriate clinical samples such as serum and tissue before administration of anti-toxin and commencement of antibiotics respectively. The early collection of samples following onset of symptoms maximises the opportunity for diagnosis. Confirmation was undertaken by either detection of toxin in serum by mouse bioassay (MBA), or by isolation of C*lostridium botulinum* from pus or wound tissue by culture and PCR detection of neurotoxin genes. Typing of C. botulinum isolates was performed by fAFLP as described previously [19].

### Police investigation

The public health investigation of this outbreak was supported by a parallel criminal investigation undertaken by Police Scotland. The objective of their investigation was to determine the source of the suspected contaminated heroin with a view to removing or reducing the amount of contaminated drugs in circulation, thereby maximising the safety of all individuals involved in the outbreak.

## Results

### Descriptive epidemiology

**Fig. 1:**
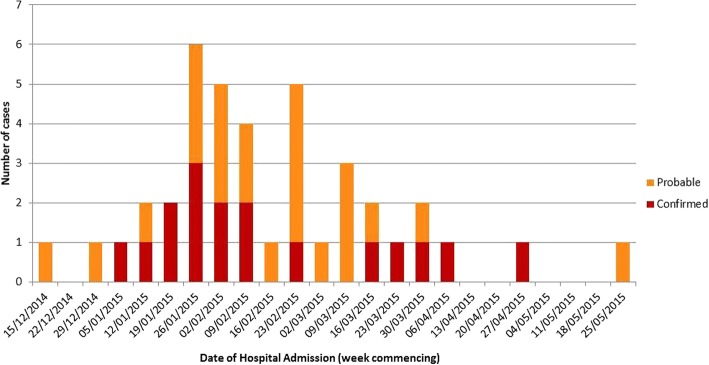
Date of hospital admission and case definitions of wound botulism infections among people who inject drugs, 21st of December 2014 to 29th of May 2015. *N* = 40

A total of 47 individuals presented to hospital, between the 21st of December 2014 and the 29th of May 2015, with symptoms indicative of botulism. Seventeen of these were confirmed microbiologically and 23 cases were classed as probable (Fig. 1). Two cases were classified as (and remain) possible, with the remaining five cases discounted. The following analysis describes the 40 confirmed/probable cases only.

The majority of cases (98%) presented with classical symptoms of oculomotor and bulbar palsy but very few with descending limb weakness; over half (58%) were ventilated for respiratory paralysis although most did not require long-term support. All cases were promptly treated with antitoxin, 50% underwent wound debridement and all received antibiotics. There were four deaths; botulism was a contributory cause in two cases (case fatality rate (CFR) = 5%) [20].

Most of the cases were male (68%). The mean age among males and females was 44 and 38 years, respectively. All confirmed/probable cases were aged between 24 and 56 years old. The majority of cases were aged between 36 and 45 years old [20].

Cases were spread across the central belt of Scotland, with the majority residing in NHS GGC (63%), followed by NHS Lanarkshire (18%), NHS Forth Valley (15%), NHS Fife (2.5%) and NHS Ayrshire & Arran (2.5%) [20].

### Risk information

Detailed information on the history of drugs taken and route of administration was available for 34 of the 40 confirmed/probable cases. Where information was available, all cases reported using heroin, either alone (52%) or in combination with another drug. Of the 29 cases who reported their length of drug use, 50% had used drugs for over 10 years. Most of the cases (62%) reported using a combination of routes for administering their drugs in the month prior to illness (intravenous, skin/muscle popping, smoking/snorting) [20].

### Microbiology

A total of 17/40 (43%) cases were confirmed microbiologically: *Clostridium botulinum* type B was detected by PCR in wound pus or tissue from 13 patients, and botulism neurotoxin type B was detected in serum of 3 patients while in a fourth patient toxin detection was confirmed but not typed. For 2 other cases, the MBA result was unconfirmed due to insufficient serum to perform the neutralisation test. Molecular typing of the organism isolated from 11 patients gave an indistinguishable fAFLP profile indicating a common source of infection. All heroin seized by Police Scotland tested negative for *Clostridium botulinum.*

### Risk management and risk communication

In the absence of intelligence on which (or how many) batches of heroin in circulation were potentially contaminated, all PWID were considered to be at risk of exposure and infection. Information from the initial cases on the source of their drugs used prior to symptoms suggested a focus on heroin obtained in or sourced via Glasgow city. Therefore, interventions to manage the incident were targeted in GGC and surrounding NHS boards.

**Fig. 2:**
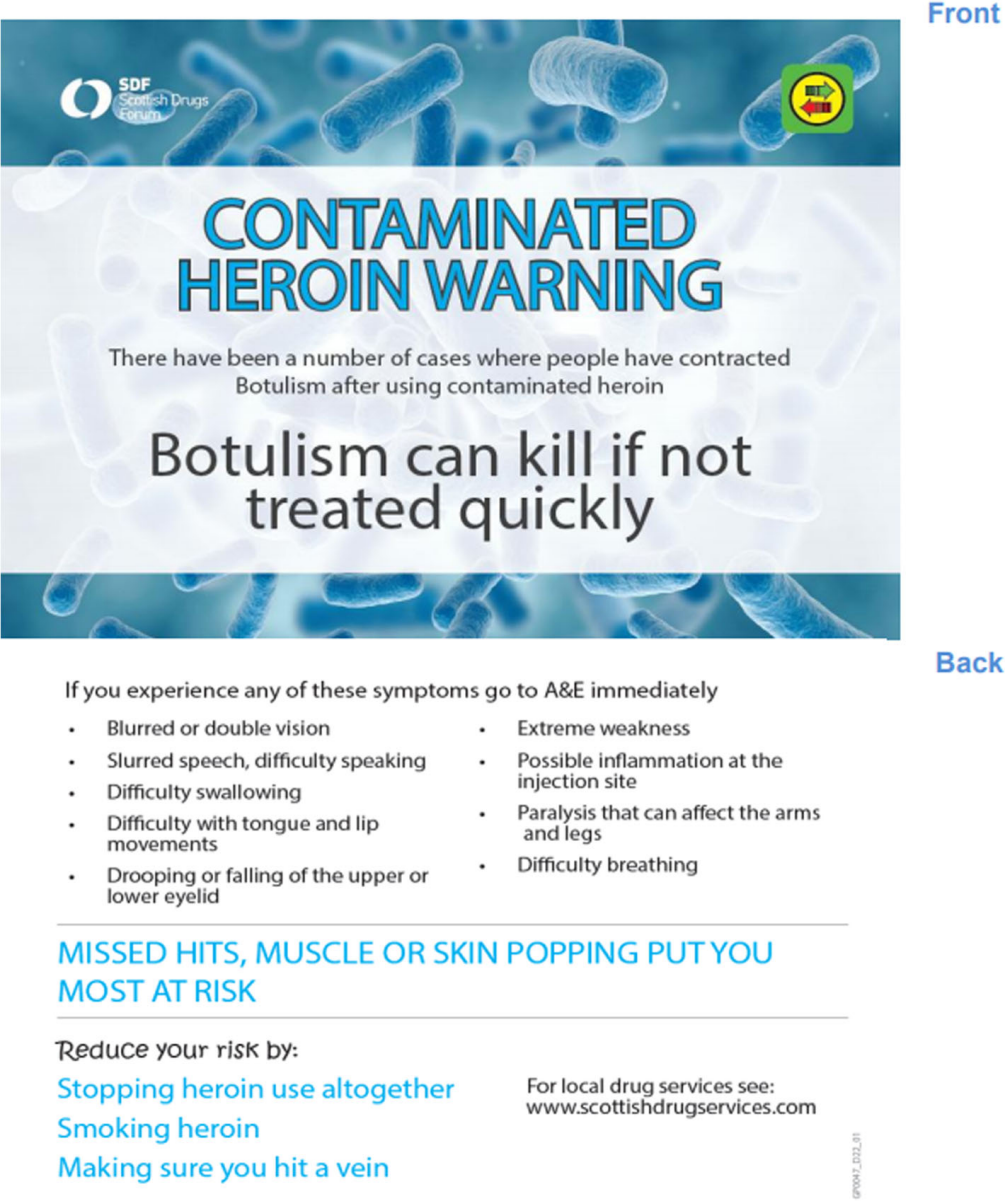
Awareness-raising postcard distributed to people who inject drugs during the outbreak

The IMT recognised that it was not a realistic expectation to be able to remove all contaminated drugs or cutting agents from the market, or to eliminate drug use among the susceptible cohort. The illicit consumption and supply of drugs mean that there is little intelligence on the source of contaminated drugs. Thus, a pragmatic harm reduction approach was adopted (Table 2). This approach, while recognising that the only way to eliminate risk of infection was to stop using drugs, also deployed interventions to reduce risk as far as possible for those who continued to inject and for those who were infected, reduce the risk of progression to serious illness. To raise awareness of the outbreak, communicate safer injection practices (for example, do not inject into the muscle or skin and promote smoking rather than injecting) and encourage early recognition of signs and symptoms, a postcard was created and made available to those at risk via all frontline and addiction services (mainly IEP services) across the NHS board areas involved (Fig. 2). The postcard was intended as an instrument for IEP staff to engage PWID in discussion, to raise awareness of botulism and safer injecting practices which reduce the risk of botulism. In addition, and in recognition that these services were key to identifying individuals at risk and filtering harm reduction messages, an information booklet, “Wound botulism and drug use: What workers need to know” composed by the Scottish Drug Forum (SDF) (available from: http://www.sdf.org.uk/wp-content/uploads/2017/03/Botulism_Booklet.pdf), was developed and distributed to frontline staff with an opportunity to reinforce the learning through a workshop delivered by SDF. Furthermore, this ensured that staff were equipped to answer questions from PWID about the outbreak.

**Table 2:** Hierarchy of objectives and advice/interventions

Objective	Advice/intervention
Eliminate risk of infection	• Advice on how to reduce or eliminate drug use altogether • Information on access to opiate substitution therapy services
Reduce risk of infection	• Advice on switching to safer route of drug use • Provision of foil to encourage smoking as an alternative to injecting • Advice on safer injecting behaviour (ensuring they inject into a vein)
Reduce risk of severity	• Education and awareness raising of the signs and symptoms of illness and where to seek help

The IMT released three media statements and communicated regularly through the duration of the outbreak with GPs, hospitals and services for drug users to raise awareness of signs and symptoms, diagnostic procedures and how to obtain botulism antitoxin.

## Discussion

A multi NHS board outbreak of *Clostridium botulinum* among PWID in Scotland resulted in 40 cases of botulism, representing the largest outbreak of wound botulism among PWID in Europe to date [18]. The epidemiology of the cases (predominantly male and over 30 years old) is representative of the PWID population in GGC [18]. Clostridium botulinum spores were not detected in samples of heroin which were obtained by Police Scotland, reflecting experiences from other outbreaks [8, 21, 22]. However, all epidemiological evidence suggested a contaminated batch of heroin or cutting agent as the source of the outbreak [20]. Given that the heroin in circulation in the UK is predominantly supplied through trafficking routes from Afghanistan, it is likely that a contamination event at some point higher up the distribution chain is the source of infections reported in the UK and Europe [2, 17].

There are significant challenges associated with managing an outbreak of botulism among PWID. The only way to completely eliminate the risk of infection is to remove all contaminated drugs from the market, or to eliminate drug use among the susceptible population, neither of which are a realistic expectation. The recommendations and interventions to minimise the risk to PWID deployed during this outbreak were by necessity pragmatic, and focused on reducing the risk of infection for those who continued to inject drugs, and limiting the consequences for those who became infected. The overall strategy to manage the outbreak was predicated on augmenting the comprehensive harm reduction services currently deployed in Scotland [23, 24], with enhanced interventions aimed at specifically reducing risk of exposure, e.g. the provision of foil to encourage smoking as an alternative to injecting, reviewing and expanding access to injecting equipment provision (IEP) and opiate substitution therapy (OST) services. While there is a lack of evidence that increasing injecting equipment provision reduces risk from bacterial infection, research suggests that it can attract and retain PWID in services [25] and therefore provide a vital window of opportunity to raise awareness of the outbreak and to filter harm reduction messages to those at ongoing risk.

The communication strategy employed during the outbreak had two strands. The first strand involved communicating with health professionals. Many frontline healthcare workers (HCW) have limited practical experience of identifying cases of botulism, especially as signs of drug overdose or effects of multiple drugs can mask early signs of botulism [7]. As early diagnosis and treatment improves outcome and reduces risk of death [26–28], the IMT alerted frontline healthcare professionals to the presenting symptoms and provided guidance on the clinical management of botulism including how to obtain botulism antitoxin. The impact of ensuring awareness of the outbreak and quicker treatment initiation was reflected in shorter hospital stays as the outbreak continued and the majority of cases making a full recovery [29]. All information resources used during the outbreak were shared widely across Scotland. In addition, given the role Glasgow plays in the distribution of drugs throughout Scotland and the potential for contaminated heroin to be re-distributed across the country or elsewhere in the Europe, all resources developed to manage the incident were shared with colleagues across the country. Furthermore, an alert notice was posted through the European Union’s (EU) Early Warning and Response System (EWRS) on 2 January 2015.

The second strand included communicating with those at risk. Experience from previous outbreaks of spore-forming bacteria in PWID suggested that there was limited benefit of using conventional press or mass media as outlets for awareness-raising activities. Information was therefore targeted at those at risk through the development of a “postcard” early in the outbreak which contained information on risk reduction measures and signs and symptoms of infection (Fig. 2). Given the sudden onset of the outbreak and the need to respond quickly, peers were not involved in the development of the postcard but reviewed the postcard after it had been created. The impact of the postcard was evaluated via the Needle Exchange Surveillance Initiative [30, 31] and qualitative interviews with persons who attended NHS GGC IEP services and had injected in the previous 6 months. The results suggested that the postcard may have improved awareness of the outbreak, the signs and symptoms of botulism and encouraged injecting behaviour change to reduce the risks of exposure, particularly when it was used as a prompt by frontline service providers for a focused discussion. The qualitative arm of the study revealed that the injection advice from the postcard which was most effective was to “ensure that they inject into a vein”. Research suggests that PWID are grateful of pragmatic injecting advice [32] which recognises that PWID will continue to inject, despite risk of infection. Further research is needed, however, to establish the best mechanism for distribution and engagement with PWID, as many remained unaware of the risk they face during an outbreak [31].

Given the special role that frontline HCWs and IEP staff have as an access point to reach PWID at risk, their views and experiences of the response and communication of the outbreak should be explored to identify barriers and ensure best practice in future outbreaks. Improving communication and trust between these service providers and peers may improve the effectiveness of future outbreak alerts among PWID [33]. Research has highlighted that communication from peer-based social networks are an important source of information [34] and should be utilised more often. Thus, peer-delivered communication strategies and the involvement of peers and frontline staff in the development of interventions such as the postcard could be key to overcoming the communication barriers experienced during this outbreak.

Officials in Glasgow have proposed the opening of a drug consumption room (DCR) and the establishment of a heroin-assisted treatment (HAT) service [35], in response to past outbreaks of spore-forming bacteria [13–15], high incidence of drug-related deaths [36] and an ongoing HIV outbreak [37]. The establishment of both these services may be an effective method of minimising the risk of another outbreak of spore-forming bacteria. HAT services would usually have stringent inclusion criteria [38]; however, these criteria could be relaxed during a severe outbreak. Furthermore, a DCR could promote safer injecting practices [39], potentially reducing the probability of soft tissue infections and therefore reducing the risk of wound botulism. Additionally, the presence of HCW in the DCR may help the detection and early recognition of spore-forming bacterial infections.

## Conclusion

The management of this outbreak, the largest to date in Europe [18], highlights the importance and need for greater recognition of a pragmatic harm reduction approach that supports the addiction needs of those vulnerable to infection during an outbreak of spore-forming bacteria. The approach adopted took onboard significant experiential learning that had been gained from similar outbreaks of spore-forming bacterial infection in the UK since 2000 [5, 13–17]. Alongside expert knowledge and published evidence where available, the experimental learning has been collated into a single guidance document published by the Scottish Health Protection Network “Guidelines for the public health management of tetanus, botulism or anthrax among people who use drugs” [40], to facilitate an efficient and effective response to future outbreaks. The response to this outbreak could have been further improved through deeper collaborations with peer and frontline staff, particularly by considering the use of peer-based social networks to filter harm reduction communications.

## References

[1] Sobel J (2005). Botulism. Clin Infect Dis.

[2] Hope VD, Palmateer N, Wiessing L, Marongiu A, White J, Ncube F, Goldberg D (2012). A decade of spore-forming bacterial infections among European injecting drug users: pronounced regional variation. Am J Public Health.

[3] Gordon RJ, Lowy FD (2005). Bacterial infections in drug users. N Engl J Med.

[4] Passaro DJ, Werner SB, McGee J, Mac Kenzie WR, Vugia DJ (1998). Wound botulism associated with black tar heroin among injecting drug users. JAMA.

[5] Jones JA, Salmon JE, Djuretic T, Nichols G, George RC, Gill ON (2002). An outbreak of serious illness and death among injecting drug users in England during 2000. J Med Microbiol.

[6] Hope VD, Hickman M, Parry JV, Ncube F (2014). Factors associated with recent symptoms of an injection site infection or injury among people who inject drugs in three English cities. Int J Drug Policy.

[7] Wenham TN (2008). Botulism: a rare complication of injecting drug use. Emerg Med J.

[8] Werner SB, Passaro D, McGee J, Schechter R, Vugia DJ (2000). Wound botulism in California, 1951–1998: recent epidemic in heroin injectors. Clin Infect Dis.

[9] MacDonald E, Arnesen TM, Brantsaeter AB, Gerlyng P, Grepp M, Hansen BÅ, Jønsrud K, Lundgren B, Mellegård H, Møller-Stray J, Rønning K (2013). Outbreak of wound botulism in people who inject drugs, Norway, October to November 2013. Eur Secur.

[10] Alpers K, van Treeck U, Frank C (2005). Outbreak of wound botulism in injecting drug users in Germany, October-December 2005. Eur Secur.

[11] Akbulut D, Dennis J, Gent M, Grant K, Hope V, Ohai C, McLauchlin J, Mithani V, Mpamugo O, Ncube F, Souza-Thomas D (2005). Wound botulism in injectors of drugs: upsurge in cases in England during 2004. Eur Secur.

[12] Schroeter M, Alpers K, van Treeck U, Frank C, Rosenkoetter N, Schaumann R (2009). Outbreak of wound botulism in injecting drug users. Epidemiol Infect.

[13] McGuigan CC, Penrice GM, Gruer L, Ahmed S, Goldberg D, Black M, Salmon JE, Hood J (2002). Lethal outbreak of infection with Clostridium novyi type A and other spore-forming organisms in Scottish injecting drug users. J Med Microbiol.

[14] Hahné SJ, White JM, Crowcroft NS, Brett MM, George RC, Beeching NJ, Roy K, Goldberg D (2006). Tetanus in injecting drug users, United Kingdom. Emerg Infect Dis.

[15] Ramsay CN, Stirling A, Smith J, Hawkins G, Brooks T, Hood J, Penrice G, Browning LM, Ahmed S (2010). An outbreak of infection with Bacillus anthracis in injecting drug users in Scotland. Eur Secur.

[16] Booth MG, Hood J, Brooks TJ, Hart A (2010). Anthrax infection in drug users. Lancet.

[17] Palmateer NE, Hope VD, Roy K, Marongiu A, White JM, Grant KA, Ramsay CN, Goldberg DJ, Ncube F (2013). Infections with spore-forming bacteria in persons who inject drugs, 2000–2009. Emerg Infect Dis.

[18] Public Health England, Health Protection Scotland, Public Health Wales, and Public Health Agency Northern Ireland (2016). Shooting up: infections among people who inject drugs in the UK, 2015.

[19] Desai M, Logan JM, Frost JA, Stanley J (2001). Genome sequence-based fluorescent amplified fragment length polymorphism of Campylobacter jejuni, its relationship to serotyping, and its implications for epidemiological analysis. J Clin Microbiol.

[20] Health Protection Scotland. Incident Management Team Report. Botulism among people who inject drugs in Scotland, December 2014 to July 2015. http://www.hps.scot.nhs.uk/resourcedocument.aspx?id=6002. Accessed 5 Apr 2018.

[21] Barry J, Ward M, Cotter S, MacDiarmada J, Hannan M, Sweeney B, Grant KA, McKeown P. Botulism in injecting drug users, Dublin, Ireland, November-December 2008. Eurosurveillance. 2009;14(1):19082. 19161713

[22] McLauchlin J, Mithani V, Bolton FJ, Nichols GL, Bellis MA, Syed Q, Thomson RP, Ashton JR (2002). An investigation into the microflora of heroin. J Med Microbiol.

[23] Information Services Division (ISD) (2016). Injecting Equipment Provision in Scotland 2014/15.

[24] Scottish Government (2010). Guidelines for services providing injecting equipment: best practice recommendations for commissioners and injecting equipment provision (IEP) services in Scotland.

[25] European Centre for Disease Prevention and Control (2011). Prevention and control of infectious diseases among people who inject drugs.

[26] Offerman SR, Schaefer M, Thundiyil JG, Cook MD, Holmes JF (2009). Wound botulism in injection drug users: time to antitoxin correlates with intensive care unit length of stay. West J Emerg Med.

[27] Chalk C, Benstead TJ, Keezer M. Medical treatment for botulism. Cochrane Database Syst Rev. 2011(3):CD008123. 10.1002/14651858.CD008123.pub2 21412916

[28] Chang GY, Ganguly G (2003). Early antitoxin treatment in wound botulism results in better outcome. Eur Neurol.

[29] Martin SJ, Penrice G, Amar C, Grant K, Gorrie GH. Wound botulism, its neurological manifestations, treatment and outcomes: a case series from the Glasgow outbreak, 2015. Scott Med J. 2017; 10.1177/0036933017707165. 10.1177/0036933017707165 28480790

[30] Health Protection Scotland (2017). Needle Exchange Surveillance Initiative: Prevalence of blood-borne viruses and injecting risk behaviours among people who inject drugs attending injecting equipment provision services in Scotland, 2008–09 to 2015–16.

[31] Dunleavy K, Munro A, Roy K, Hutchinson S, Palmateer N, Knox T, Goldberg D, Hope V, Campbell J, Hamilton E, Liddell D (2018). Spore forming bacteria infections and people who inject drugs: implications for harm reduction. Int J Drug Policy.

[32] Harris M, Rhodes T (2012). Venous access and care: harnessing pragmatics in harm reduction for people who inject drugs. Addiction.

[33] Soukup-Baljak Y, Greer AM, Amlani A, Sampson O, Buxton JA (2015). Drug quality assessment practices and communication of drug alerts among people who use drugs. Int J Drug Policy.

[34] Markwick N, McNeil R, Anderson S, Small W, Kerr T (2016). Communicating risk in the context of methadone formulation changes: a qualitative study of overdose warning posters in Vancouver, Canada. Int J Drug Policy.

[35] Tweed E, Rodgers M (2016). Taking away the chaos the health needs of people who inject drugs in public places in Glasgow city centre.

[36] National Records of Scotland (2017). Statistics of drug-related deaths in 2016 and earlier years, broken down by age, sex, selected drugs reported, underlying cause of death and NHS Board and Council areas.

[37] Ragonnet-Cronin M, Jackson C, Bradley-Stewart A, Aitken C, McAuley A, Palmateer N, Gunson R, Goldberg D, Milosevic C, Leigh Brown AJ (2018). Recent and rapid transmission of HIV among people who inject drugs in Scotland revealed through phylogenetic analysis. J Infect Dis.

[38] Strang J, Groshkova T, Uchtenhagen A, van den Brink W, Haasen C, Schechter MT, Lintzeris N, Bell J, Pirona A, Oviedo-Joekes E, Simon R (2015). Heroin on trial: systematic review and meta-analysis of randomised trials of diamorphine-prescribing as treatment for refractory heroin addiction. Br J Psychiatry.

[39] Stoltz JA, Wood E, Small W, Li K, Tyndall M, Montaner J, Kerr T (2007). Changes in injecting practices associated with the use of a medically supervised safer injection facility. J Public Health.

[40] Scottish Health Protection Network (2017). Guidelines for the public health management of tetanus, botulism or anthrax among people who use drugs.

